# Insights into the Structure-Activity Relationship of Glycosides as Positive Allosteric Modulators Acting on P2X7 Receptors[Fn FN3]

**DOI:** 10.1124/molpharm.120.000129

**Published:** 2021-02

**Authors:** Waraporn Piyasirananda, Andrew Beekman, A. Ganesan, Stefan Bidula, Leanne Stokes

**Affiliations:** School of Pharmacy, University of East Anglia, Norwich Research Park, Norwich, United Kingdom

## Abstract

P2X7 is an important ligand-gated ion channel expressed in multiple immune cell populations. This study aimed to investigate the chemical requirements of triterpenoid glycosides within a new binding pocket to characterize the structure-activity relationship. A set of glycosides were screened for positive modulator activity at human P2X7 using a YO-PRO-1 dye uptake assay in HEK-293 cells stably expressing the wild-type human P2X7 variant (HEK-hP2X7 cells). The highest positive modulator activity was with ginsenoside–compound K (CK), containing a monosaccharide (glucose) attached at carbon-20. Ginsenoside-20(*S*)-Rg3, containing a disaccharide group (glucose-glucose) at carbon-3, displayed positive modulator activity with a reduced EC_50_ for ATP and increased maximal response at human P2X7. The epimer 20(*R*)-Rg3 was inactive. A similar stereo-specific pattern was observed for 20(*S*)-Rh2. Ginsenoside-F1, highly similar to ginsenoside-CK but containing a single additional hydroxyl group, was also inactive at P2X7. Computational docking suggests hydrophobic residues in the pocket are involved in steric discrimination between triterpenoids, whereas the position and identity of the carbohydrate group are important for positive modulator activity at human P2X7. Ginsenosides containing monosaccharide attachments perform better than di- or trisaccharide glycosides. Additional modifications to the triterpenoid scaffold at carbon-6 are not tolerated. Gypenosides from plant sources other than *Panax ginseng* (gypenoside XVII, gypenoside XLIX, stevenleaf) can also act as positive allosteric modulators of P2X7. We also investigated the effect of positive allosteric modulators on endogenous P2X7 in THP-1 monocytes and confirmed our findings in a calcium response assay. A cell viability assay showed potentiation of ATP-induced cell death with ginsenoside-CK in THP-1 and HEK-hP2X7 cells.

**SIGNIFICANCE STATEMENT:**

Ginsenosides are active as positive allosteric modulators at P2X7, and this study determines the chemical features important for mediating this effect. The position and identity of the sugar group is important for activity, as is the position of a number of hydroxyl groups on the triterpenoid scaffold. Diastereomers of ginsenoside-Rg3 and ginsenoside-Rh2 demonstrate the importance of the location of hydroxyl groups relative to the hydrophobic face of the predicted binding pocket.

## Introduction

The ligand-gated ion channel P2X7 is important in regulating immune cell responses during infection and inflammation (Di Virgilio et al., 2017). In particular, P2X7 is a known physiologic regulator of the NLRP3–caspase 1 inflammasome complex and controls the secretion of proinflammatory cytokines such as interleukin-1*β* and interleukin-18 (Giuliani et al., 2017). Many studies have demonstrated that activation of P2X7 in infected macrophages in vitro can promote microbial killing. Intracellular bacteria and parasites such as *Mycobacterium tuberculosis* (Fairbairn et al., 2001, Placido et al., 2006), *Toxoplasma gondii* (Moreira-Souza et al., 2017), and *Leishmania amazonensis* (Chaves et al., 2014) are examples of pathogens in which P2X7 contributes to microbicidal mechanisms. In vivo mouse models of infection suggest that global deficiency in P2X7 can affect pathogen burden and inflammation (Miller et al., 2015; Chaves et al., 2019). Furthermore, inheritance of loss-of-function variants of human P2X7 have been linked to susceptibility to infections and complications such as extrapulmonary tuberculosis (Fernando et al., 2007; Areeshi et al., 2015). Development of a positive allosteric modulator (PAM) of P2X7 may therefore be useful in treatment of such infections (Stokes et al., 2020). This type of therapy directed at enhancing host responses would reduce the need for antibiotics and be beneficial in avoiding development of antibiotic resistance.

Triterpenoid glycosides from *Panax ginseng* have PAM activity at human P2X7 with the in vivo metabolite ginsenoside–compound K (CK) demonstrating the highest activity (Helliwell et al., 2015). Using a combination of computational docking and mutagenesis, we previously characterized a novel binding pocket for ginsenosides within the central vestibule of human P2X7 (Bidula et al., 2019b). Predicted binding poses identified two modes of binding dependent on the carbohydrates attached to the ginsenoside in question. Ginsenoside-CK contains a single carbohydrate (glucose) moiety that makes multiple contacts with amino acids Asp318, Leu320 and Ser60. Conversely, ginsenoside-Rd contains both disaccharide (-glucose-glucose) and monosaccharide glucose moieties and is predicted to use the disaccharide moiety for binding to P2X7 (Bidula et al., 2019b). The presence of carbohydrate moieties is deemed essential for PAM activity since the aglycone protopanaxadiol has no activity at P2X7 (Helliwell et al., 2015).

In this study, we explored the chemical structural requirements of triterpenoid glycosides with the aim of building a structure-activity relationship for positive modulators at P2X7. This revealed critical information about the tolerance of substitutions at carbon (C)-6 and the preference for glucose as the attached sugar moiety. This provides important knowledge for the future development of selective PAMs for P2X7.

## Materials and Methods

### 

#### Materials.

Ginsenoside-CK (CAS 39262-14-1), ginsenoside-Rb1 (CAS 41753-43-9), ginsenoside-Rd (CAS 52705-93-8), 20(*S*)-ginsenoside-Rg3 (CAS 14197-60-5), 20(*R*)-ginsenoside-Rh2 (CAS 112246-15-8), protopanaxadiol (PPD) (CAS 30636-90-9), glycyrrhizic acid (CAS 1405-86-3), stevioside (CAS 57817-89-7), daucosterol (CAS 474-58-8), esculentoside A (CAS 65497-07-6), mogroside V (CAS 88901-36-4), saikosaponin A (CAS 20736-09-8), and stevenleaf (CAS 80321-63-7) were from Shanghai Richem International Ltd., China (supplier code CDCMANSETE). Ginsenoside-CK (CAS 39262-14-1), ginsenoside-F2 (CAS 62025-49-4), ginsenoside-F1 (CAS 53963-43-2), gypenoside XLIX (CAS 94987-08-3), gypenoside XVII (CAS 80321-69-3), 20(*S*)-ginsenoside-Rg3 (CAS 14197-60-5), 20(*R*)-ginsenoside-Rg3 (CAS 38243-03-7), 20(*S*)-ginsenoside-Rh2 (CAS 78214-33-2), and 20(*R*)-ginsenoside-Rh2 (CAS 112246-15-8) were from ChemFaces (Wuhan Chemfaces). Scilliroside (CAS 507-60-8, NSC7523), ouabain (CAS 630-60-4, NSC25485), solanine hydrochloride (CAS 20562-02-1, NSC35611), and solasonine (CAS 19121-58-5, NSC82149) were obtained from the National Cancer Institute Developmental Therapeutics Program chemical repository. ATP (A7699; Sigma-Aldrich) was dissolved in distilled water to 100 mM and adjusted to pH 7.4 with 5 M NaOH. Aliquots of ATP were kept frozen at −20°C and used once in experiments. AZ10606120 (CAS 607378-18-7; Tocris Biosciences) and AZ11645373 (CAS 227088-94-0; Sigma-Aldrich) were dissolved in DMSO to 10 mM and stored at −20°C. Fura-2 acetoxymethyl ester (AM) (CAS 108964-32-5; HelloBio) was prepared in DMSO to a concentration of 1 mM, aliquoted, and stored at −20°C in amber-colored Eppendorf vials. Sulfinpyrazone (CAS 57-96-5; Sigma Aldrich) was dissolved in methanol at a stock concentration of 25 mM and stored at +4°C.

#### Cell Culture.

HEK-293 cells stably expressing the wild-type human P2X7 (hP2X7) variant (HEK-hP2X7) were maintained in Dulbecco’s modified Eagle’s medium (DMEM):F12 (catalog number 11320-074; ThermoFisher Scientific, Life Technologies) containing 10% fetal bovine serum (catalog number 10500-064; Gibco) and penicillin/streptomycin (ThermoFisher Scientific) as described previously (Helliwell et al., 2015). HEK-293 cells were passaged twice weekly using 0.25% trypsin-EDTA (ThermoFisher Scientific). THP-1 human monocytic cells (a kind gift from Professor Maria O’Connell, University of East Anglia) were maintained in RPMI 1640 medium (catalog number 21875-034, Fisher Scientific, Life Technologies) containing 10% fetal bovine serum (Gibco, as before) and penicillin/streptomycin (ThermoFisher Scientific). Cells were kept in a humidified incubator at 37°C with a constant supply of 5% CO_2_.

#### YO-PRO-1 Dye Uptake Screening Assay.

ATP-induced dye uptake experiments were performed as described previously (Bidula et al., 2019b). Briefly, HEK-hP2X7 cells were plated at 2 × 10^4^ cells per well (100 μl per well) in complete medium and left overnight to attach to poly-d-lysine–coated 96-well plates. YO-PRO-1 iodide was prepared in a low divalent buffered solution (145 mM, 2 mM KCl, 13 mM d-glucose, 10 mM HEPES, 0.2 mM CaCl_2_ pH 7.3) to a final concentration of 2 μM. Medium was removed from the plate using a manual multichannel pipette and YO-PRO-1 containing buffer applied to wells (180 μl per well). AZ10606120 (10 μM) was prepared in YO-PRO-1 low divalent buffer and added directly to the cells to block P2X7 by pretreatment. A Flexstation 3 plate reader (Molecular Devices, Sunnyvale, CA) was used to record YO-PRO-1 fluorescence in HEK-hP2X7 for 300 seconds after the addition of ATP (at 10× final concentration) with/without ginsenoside compounds or vehicle (DMSO). Plates were allowed to warm up to 37°C for 10 minutes before initiating the recording. Baseline recordings were made for 40 seconds before compound addition using the Flexstation 3 fluidics system. YO-PRO-1 fluorescence was measured at 520 nm after excitation at 490 nm (auto cutoff at 495 nm), and the sample interval was 3.5 seconds. Flexstation settings were photomultiplier tube (PMT) medium, six reads per well, pipette height 170, and a rate of injection of 3. Data were acquired using Softmax Pro version 5.4 (Molecular Devices). To analyze the data, area under the dye uptake curve was calculated in Softmax Pro version 5 using zero baseline data with a lag time of 50 seconds (50–300 seconds).

#### Fura-2 Calcium Measurements.

THP-1 cells were pelleted by centrifugation (300*g*, 5 minute), washed, and resuspended in Hanks’ balanced salt solution buffer containing 2 μM fura-2 AM (HelloBio) and 250 μM (±)-sulfinpyrazone. Cells were loaded for 1 hour in a waterbath at 37°C while shielded from light with foil. Loaded cells were washed once with 5 ml Hanks’ balanced salt solution to remove excess fura-2 AM dye. THP-1 cells were then resuspended, counted, and plated at 2 × 10^5^ cells per well in a standard clear 96-well plate (NUNC 167008; Thermo Scientific) in low divalent assay buffer without Mg^2+^ (145 mM NaCl, 2 mM KCl and 2 mM CaCl_2_, 13 mM glucose, 10 mM HEPES, pH 7.3) containing 250 μM (±)-sulfinpyrazone. The 10× concentration of agonist (ATP) was prepared in the same assay buffer. ATP was injected automatically into wells at 30 seconds in a Flexstation 3 microplate reader. Ratiometric data were acquired using 340 and 380 nm for excitation wavelengths, 520 nm as the emission wavelength, and six reads per well (PMT medium). Area under the curve was calculated using standard zero baseline normalization with a lag time 0–300 seconds using SoftMax Pro version 5.4 software.

#### Cell Viability Assay.

HEK-hP2X7 or THP-1 cells were seeded at 5 × 10^3^ cells per well in a volume of 100 μl for 24 hours in 96-well plates (Nunclon Edge plates, catalog number 167425; Thermo Scientific). Cells were plated in DMEM:F12 medium or RPMI 1640 medium containing 1% FBS and penicillin/streptomycin for 24 hours in a humidified incubator at 37°C with a constant supply of 5% CO_2_. Edge wells of the plates were filled with autoclaved distilled water to prevent evaporation of media from wells containing cells. The compounds and ATP were made at 2× final concentration in 1% FBS DMEM:F12 and 100 μl added to the cells for 24 hours. After incubation, resazurin sodium salt (CAS 62758-13-8; Sigma Aldrich) at 0.1 mg/ml in sterile PBS was added to the plate (20 into 200 µl culture medium per well). Cells were incubated for a further 2 hours in a humidified incubator at 37°C with a supply of 5% CO_2_. A Flexstation 3 microplate reader was used to acquire data. Endpoint fluorescence data were measured using 570 nm excitation wavelength, 600 nm emission wavelength (cutoff at 590 nm), and three reads per well (PMT low). Data were analyzed by performing background correction through subtracting blank medium readings (without cells) from all samples and then normalizing each sample to medium treated cells (control treatment, 100%) as percentage of control.

#### Computational Docking.

The homology model of human P2X7 generated previously in (Bidula et al., 2019b) was used for docking runs using the Schrödinger Maestro suite. Three-dimensional models of ginsenoside-F1 and ginsenoside-20(*S*)-Rg3 were generated using LigPrep software, and the OPLS3 force field was used to generate up to 32 low-energy conformers for each ligand. Induced-fit docking was performed using the automated extended sampling protocol, first performing several initial docking runs in which either side chains were trimmed or van der Waals potentials were softened according to their flexibility; then side chains were rebuilt, and those within 5 Å of the ligand were optimized using Prime software (Jacobson et al., 2004). Structures within 30 kcal mol^−1^ of the lowest energy structure were retained. Ligands were then redocked to the new receptor structure using the Glide SP algorithm (Friesner et al., 2004) and standard potentials. The receptor grid was centered on the highest scoring potential binding site using SiteMap (Halgren, 2007), and this had a cubic box with dimensions of 30 Å. For each ginsenoside the resulting poses were clustered by heavy atom root-mean-square deviation using the average-linkage method, and a representative structure was chosen from the model closest to the centroid of the most populated cluster. For ginsenoside-F1 and 20(*S*)-Rg3 the most populated cluster made up 31% and 62% of all solutions, respectively.

#### Data and Statistical Analysis.

Results are expressed as means ± S.D. from the indicated number of experiments. For YO-PRO-1 dye uptake and intracellular calcium experiments, each independent experiment used triplicate wells, and the means of the replicates were collated and plotted. Technical replicates were used to ensure reliability of fluorescence values. Cell viability data were normalized to the vehicle control for each experiment after background correction had been performed. Dose-response curves were plotted by a nonlinear regression fit with variable slope using GraphPad Prism software version 7. Half-maximal responses are expressed as EC_50_ values with 95% confidence intervals. These values were calculated from the collated data for each compound from three independent experiments. Statistical differences were determined by analysis of the data by one-way ANOVA followed by Dunnett’s multiple comparison test or Sidak’s multiple comparison test using GraphPad Prism version 7. *P* < 0.05 was the accepted minimum level of significance.

## Results

**Fig. 1.**
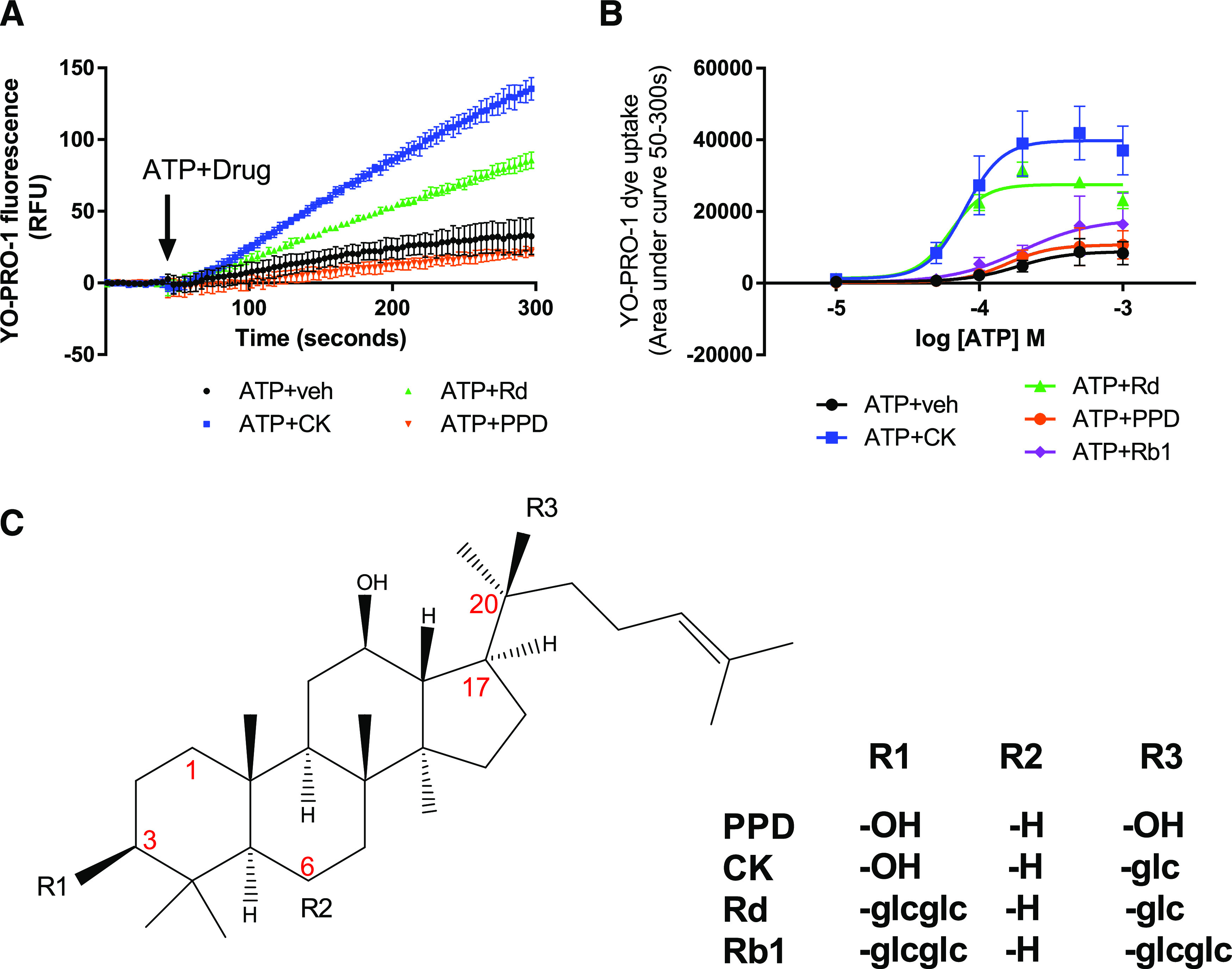
Positive allosteric effects of protopanaxadiol ginsenosides on P2X7. (A) A YO-PRO-1 iodide uptake assay was used to determine the effect of multiple ginsenosides on human P2X7 stably expressed in HEK-293 cells. Agonist (ATP, 200 μM) and modulator (10 µM) or vehicle (veh) were premixed at 10× final concentration and auto-injected together (coinjection) using a Flexstation 3 multimode plate reader. YO-PRO-1 dye uptake (relative fluorescence units (RFU)) was measured over 300 seconds. Data are expressed as area under curve means ± S.D. (B) Dose-response curves to ATP in the absence and presence of 10 µM ginsenoside-CK, -Rd, or -Rb1 or the aglycone PPD with a four-parameter nonlinear regression. Data are collated from three independent experiments each performed in triplicate. Error bars represent S.D. (C) Chemical structures for each of the ginsenoside positive modulators and the inactive aglycone PPD are shown.

To measure positive modulator activity at human P2X7 receptors, we used a well characterized HEK-hP2X7 stable cell line (Helliwell et al., 2015) and a YO-PRO-1 dye uptake assay performed on a Flexstation 3 plate reader. Figure 1A shows a typical dye uptake response to an approximate EC_50_ concentration of agonist (200 μM ATP) and the effect of coadministration of ginsenoside-CK or ginsenoside-Rd at a final concentration of 10 μM. We previously demonstrated that pretreatment with a selective P2X7 antagonist AZ10606120 abolished the response, confirming that the effect of the ginsenosides was dependent on P2X7 activation (Helliwell et al., 2015). Dose-response experiments demonstrated that ginsenoside-CK and ginsenoside-Rd have two effects on ATP-induced responses at hP2X7 (Fig. 1B): an increase in the maximum response (a type I PAM effect) and a shift in the dose-response curve to the left (a type II PAM effect), thereby enhancing the maximum effect of the agonist and reducing the EC_50_ value respectively. Classification of type I, type II and type I/II mixed PAM effects have been previously used for *N*-methyl-d-aspartate (NMDA) receptors (Hackos and Hanson, 2017), and the same naming convention for P2X receptors is discussed in a recent review (Stokes et al., 2020). Ginsenoside-CK reduced the EC_50_ for ATP to 61.6 μM compared with an average EC_50_ for ATP + vehicle (DMSO) of 219 μM (*n* = 5 experiments) and increased the maximum response by 2.4–4.4-fold (Table 1). Both ginsenoside-CK and ginsenoside-Rd have mixed type I/II effects. Ginsenoside-Rb1 increased the maximum response by 1.9-fold but had little effect on the EC_50_ value (175.9 μM; Fig. 1B) and therefore has only type I PAM activity. The aglycone ginsenoside metabolite PPD had no effect on the ATP dose-response curve (Fig. 1B).

### 

#### Investigating Glycosylation Patterns.

**Fig. 2.**
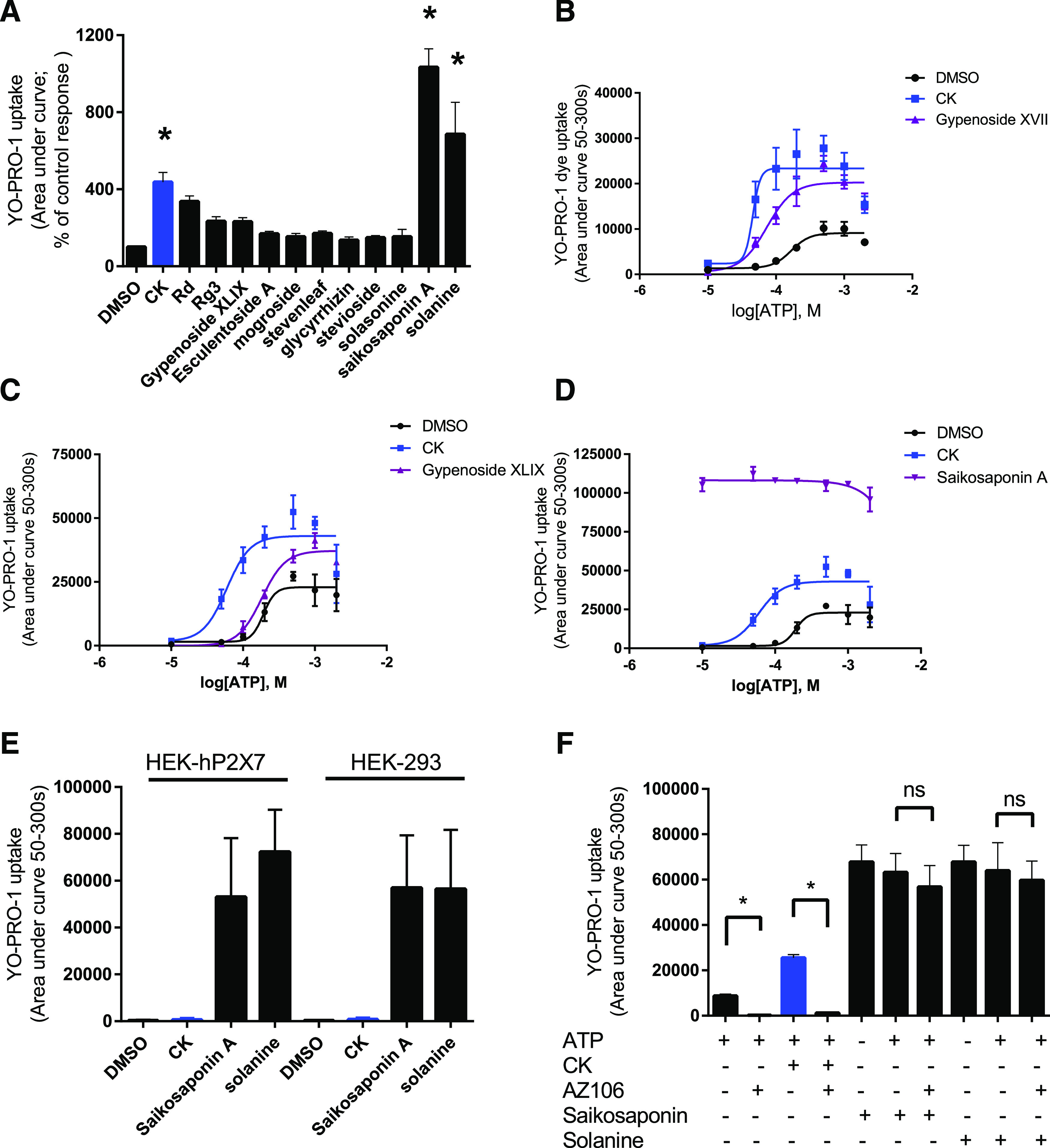
Screening glycosides containing disaccharide and trisaccharide moieties at P2X7. (A) Initial experiments used a fixed concentration of ATP (200 µM final) and glycoside (10 µM final) to screen selected glycosides at P2X7. Data are collated from two to four independent experiments. Ginsenoside-CK was used as the control PAM and is shown in blue. YO-PRO-1 uptake was measured as area under curve (50–300 seconds), and data are expressed as percentage of control, where the control is ATP + DMSO. One-way ANOVA with Dunnett’s multiple comparisons test was performed. *P < 0.05 compared with DMSO control. (B) Dose-response curve to ATP in the presence of vehicle (DMSO), ginsenoside-CK, or gypenoside XVII (10 µM). (C) Dose-response curve to ATP in the presence of vehicle (DMSO), ginsenoside-CK, or gypenoside XLIX (10 µM). (D) Dose-response curve to ATP in the presence of vehicle (DMSO), ginsenoside-CK, or saikosaponin A (10 µM). Data points are means ± S.D. The same curves from DMSO and ginsenoside-CK are shown in (B and D). (E) Summary of data from YO-PRO-1 uptake experiments in HEK-hP2X7 cells or parental nontransfected HEK-293 cells in response to drug alone (saikosaponin A or solanine). (F) Lack of effect of the P2X7-selective antagonist AZ10606120 (AZ106; 10 µM) on ATP-induced YO-PRO-1 uptake when solanine or saikosaponin were used. One-way ANOVA with Sidak’s multiple comparisons test was performed. *P < 0.05, ns denotes not significant.

We assumed that the carbohydrate groups play a key role in mediating this response at P2X7 since the aglycone PPD had no effect (Fig. 1B). Our previous study identified a binding site within the human P2X7 trimeric structure based on computational docking to a homology model. The single glucose moiety on ginsenoside-CK makes predicted interactions with P2X7 *β*-strands lining the lateral portals which connect the agonist-binding site to the ion channel transmembrane domains (Bidula et al., 2019b). We investigated the effect of varying the number of sugar moieties attached to the steroid-like scaffold by searching for chemicals similar to ginsenosides that were commercially available as purified chemicals. We excluded ginsenosides from the protopanaxatriol series with sugars attached on C-6 as our previous work has demonstrated these compounds to have no activity at P2X7 (Helliwell et al., 2015). We identified a number of candidate glycosides to test (Table 1) and screened these compounds at a final concentration of 10 μM on hP2X7 responses using a fixed concentration of ATP (200 μM) in the YO-PRO-1 dye uptake assay. Focusing on glycosides containing a disaccharide moiety first (Fig. 2) we found that ginsenoside-Rg3 (mixed isomers) and gypenoside XLIX showed a small increase in the ATP-induced YO-PRO-1 response, which was not statistically significant (Fig. 2A), whereas saikosaponin A and solanine both showed a large increase in the ATP-induced YO-PRO-1 dye uptake response (Fig. 2A). Most other glycosides in this category showed no modulation of P2X7 (esculentoside A, mogroside V, glycyrrhizic acid, and stevioside). Stevenleaf showed a minor modulation increasing the maximum response only (1.64-fold; Supplemental Fig. 1). Dose-response experiments demonstrated that gypenoside XVII has a type II PAM activity at P2X7 (Fig. 2B), reducing the EC_50_ value for ATP (Table 1) and increasing the maximum response by 2-fold. In contrast, gypenoside XLIX did not reduce the EC_50_ value for ATP (Table 1) but did increase the maximum response by 1.9-fold (Fig. 2C). Further investigations into saikosaponin A (Fig. 2D) revealed that this glycoside increased YO-PRO-1 uptake at all concentrations of ATP tested and could induce YO-PRO-1 uptake in nontransfected HEK-293 cells (which have no expression of P2X7) and in HEK-hP2X7 cells in the absence of ATP (Fig. 2E), suggesting that this effect was not P2X7-dependent. We confirmed this by pretreating HEK-hP2X7 cells with a P2X7-selective antagonist, AZ10606120. ATP-induced responses were abolished in cells treated with 10 μM AZ10606120, as were responses induced by ATP + ginsenoside-CK (Fig. 2F). However, responses to ATP + saikosaponin A or ATP + solanine were unaffected by AZ10606120 pretreatment, suggesting that P2X7 was not involved (Fig. 2F).

**TABLE 1:** Structural details of tested glycosides Substitution pattern is derived from Fig. 1C. Maximum response was defined at 1 mM ATP. To calculate the EC_50_ ratio, the average EC_50_ value for ATP + vehicle was used from each set of experiments. Average EC_50_ value is shown with 95% confidence intervals in parentheses.

Glycoside	ATP EC_50_ (μM)	EC_50_ ratio	Maximum response (fold increase)	Substitution pattern		
				R1 (C-3)	R2 (C-6)	R3 (C-20)
Ginsenoside-CK	61.6 (43.1 to 80.1) *	2.7	2.4–4.4	-OH		-glc
Ginsenoside-F1	182.3 (136.8 to 227.9)	1.1	1.46	-OH	-OH	-glc
Ginsenoside-F2	35.3 (15.1 to 55.4) *	5.4	2.23	-glc		-glc
Ginsenoside-20(*S*)-Rh2	80.0 (54.0 to 105.9) *	1.8	2.6	-glc		-OH
Ginsenoside-20(*R*)-Rh2	169.7 (110.4 to 228.9)	0.8	1.53	-glc		-OH
Daucosterol	258.5 (172.6 to 344.4)	0.7	1.33	-glc		
Ouabain	133.4 (102.8 to 163.9)	1.1	1.08	-rha		butyrolactone
Scilliroside	112.6 (78.2 to 147.1)	1.3	1.07	-glc	-OAc	2-pyrone
Stevenleaf (gypenoside IX)	184.2 (71.2 to 297.1)	0.7	1.64	-glc		-glc-xyl
Gypenoside XVII	79.9 (56.0 to 103.8) *	2.4	2.0	-glc		-glc-glc
Esculentoside A	165.5 (85.2 to 245.8)	0.8	0.94	-xyl-glc		-COOMe
Ginsenoside-20(*S*)-Rg3	78.7 (53.6 to 103.8) *	1.8	2.4	-glc-glc		-OH
Ginsenoside-20(*R*)-Rg3	146.0 (116.0 to 176.0)	1.0	0.94	-glc-glc		-OH
Ginsenoside-Rd	57.7 (42.4 to 72.9) *	3.8	2.77	-glc-glc		-glc
Ginsenoside-Rb1	175.9 (55.6 to 296.2)	1.2	1.96	-glc-glc		-glc-glc
Stevioside	189.6 (141.2 to 238.0)	1.0		-glc		-glc-glc
Glycyrrhizic acid	n.d	n.d	n.d	-glcA-glcA		-COOH
Saikosaponin A	N.A	n.d	N.A	-fuc-glc		
Gypenoside XLIX	213.1 (209.3 to 216.9)	0.9	1.9	-rha-ara-xyl		-glc
Mogroside V	n.d	n.d	n.d	-glc-glc		-glc-glc-glc
Solasonine	N.A	n.d	N.A	-rha-gal-glc		
Solanine	N.A	n.d	N.A	-rha-gal-glc		

N.A., not applicable; n.d., not determined. Carbohydrate groups: -ara; arabinose, -fuc; fucose, -gal; galactose, -glc; glucose, -glcA; glucuronic acid, -rha; rhamnose, -xyl; xylose.

*P < 0.05 from one-way ANOVA with Dunnett’s multiple comparisons test.

**Fig. 3.**
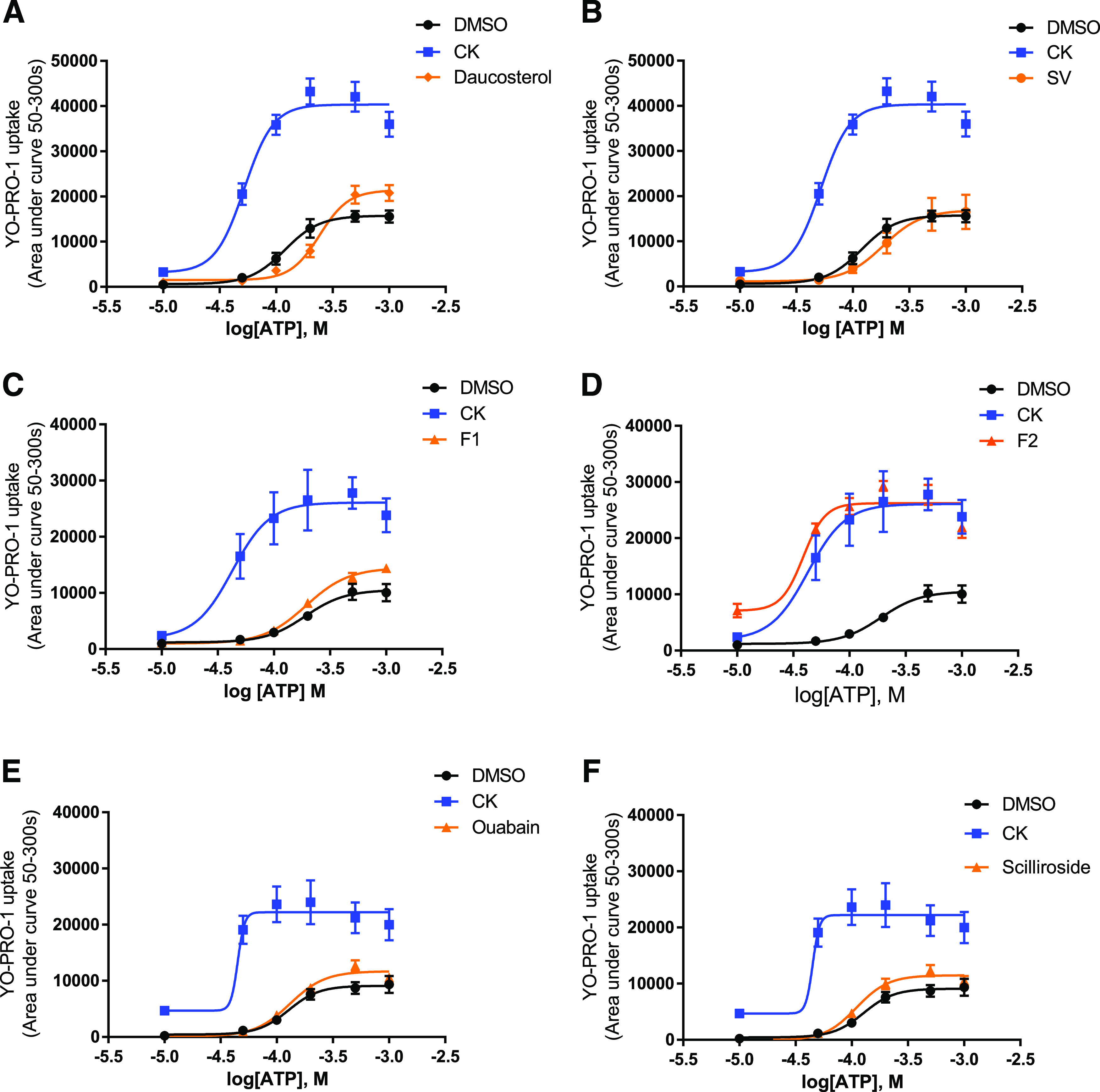
Screening glycosides containing monosaccharide moieties at P2X7. Dose-response curves to ATP in the presence of vehicle (DMSO), ginsenoside-CK, and the following: daucosterol (10 µM) (A), stevioside (SV; 10 µM) (B), ginsenoside-F1 (F1; 10 µM) (C), ginsenoside-F2 (F2; 10 µM) (D), ouabain (10 µM) (E), or scilliroside (10 µM) (F). Ginsenoside-CK is demonstrated throughout as the reference compound (same data shown in each plot). Data are collated from three independent experiments each performed in triplicate. Error bars represent S.D.

We then investigated glycosides with monosaccharide attachments including daucosterol, ginsenoside-F2, ginsenoside-F1, ouabain, and scilliroside (Table 1). Dose-response experiments demonstrated that ginsenoside-F2 could both increase the maximum response and reduce the EC_50_ value for ATP (Table 1) and displayed a similar mixed type I/II effect as ginsenoside-CK (Fig. 3D). Ouabain, daucosterol, and scilliroside had no effect on the ATP dose-response curves (Fig. 3). Interestingly, ginsenoside-F1, which has an almost identical chemical structure to ginsenoside-CK, did not potentiate P2X7 responses (Fig. 3C). The presence of one additional hydroxyl group on C-6 is the only difference between ginsenoside-CK and ginsenoside-F1, and this could potentially interfere with correct positioning into the P2X7 binding pocket.

#### Computational Docking.

**Fig. 4.**
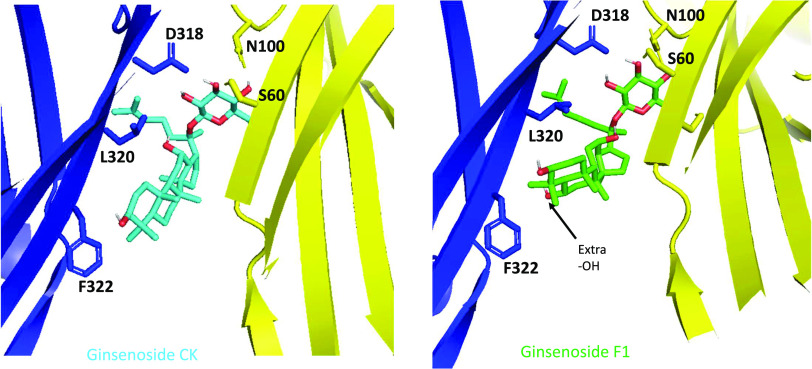
Molecular docking of ginsenoside-CK and ginsenoside-F1 to the central vestibule pocket of hP2X7. Ginsenoside-CK (cyan) docked into the central vestibule in the ATP-bound homology model of human P2X7 and right, ginsenoside-F1 (green) docked into the same site. Side chains of amino acid residues of hP2X7 implicated in interactions are shown.

To investigate the predicted theoretical docking differences between ginsenoside-CK and ginsenoside-F1, we used computational docking to a homology model of human P2X7 (Bidula et al., 2019b). Replacing ginsenoside-CK (Fig. 4A) in the open ATP-bound model of hP2X7 with ginsenoside-F1 resulted in an analogous predicted pose (Fig. 4B) and this would orient the additional hydroxyl group on C-6 to be facing the hydrophobic side of the pocket close to L320 and F322. Since ginsenoside-F1 has no activity at P2X7, this purely theoretical pose suggests that steric hindrance and a repulsive effect would prevent ginsenoside-F1 from interacting with the PAM site at P2X7. Furthermore, there would likely be an energy penalty due to poor solvation of the additional OH group within the hydrophobic pocket.

**Fig. 5.**
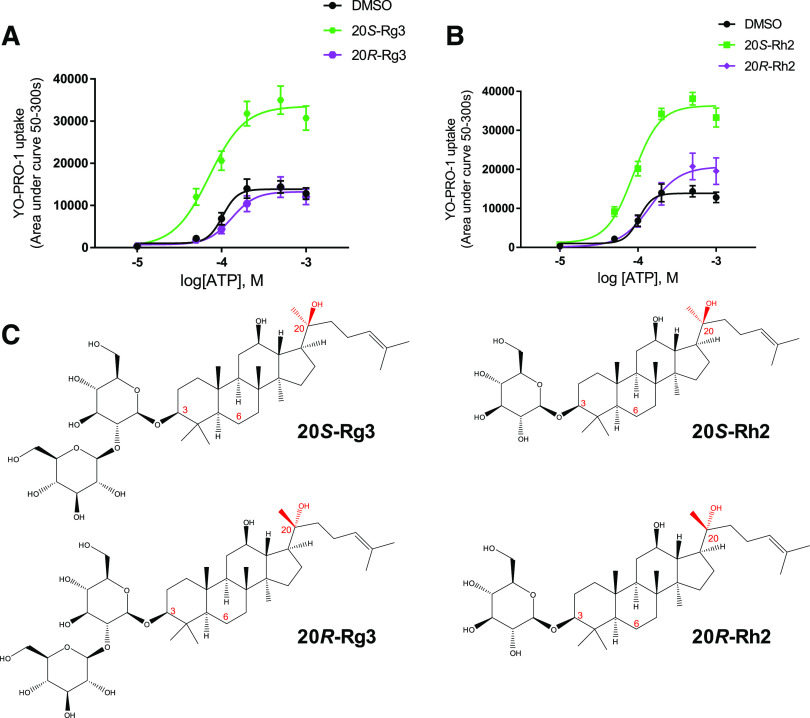
Diastereoisomers of ginsenosides have different activity at hP2X7. (A) Dose-response curves to ATP in the presence of vehicle (DMSO), 20(*S*)-ginsenoside-Rg3, or 20(*R*)-ginsenoside-Rg3 (10 µM). (B) Dose-response curves to ATP in the presence of vehicle (DMSO), ginsenoside-20(*S*)-Rh2, or ginsenoside-20(*R*)-Rh2 (10 µM). Data are collated from three independent experiments and are means ± S.D. The same DMSO data are shown in both plots. (C) Chemical structures of ginsenoside-Rg3 and ginsenoside-Rh2 are shown with the stereo-centers highlighted in red.

**Fig. 6.**
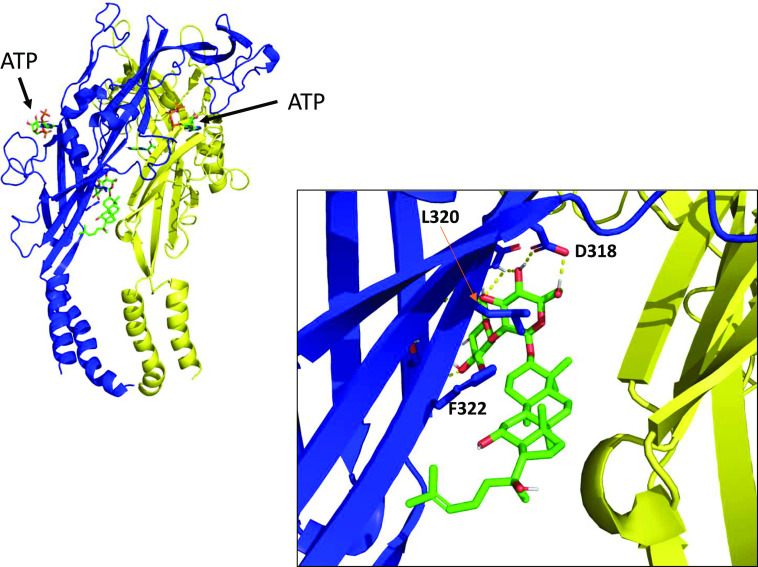
Induced-fit docking of 20S-Rg3 at human P2X7 ginsenoside-20(*S*)-Rg3 (green) docked into the central vestibule site in a homology model of ATP-bound human P2X7 (open state). The predicted orientation of the stereocentre C-20 is such that the –OH is pointing away from the hydrophobic face of the binding site, thus minimizing any repulsive interactions. Key side chains of residues D318, L320, and F322 are indicated.

Concurrently we investigated chemicals thought to bind in an inverted mode such as ginsenoside-Rd (Bidula et al., 2019b). Ginsenosides with sugars attached to C-3 rather than C-20 are predicted to insert sugars deep into the pocket and would have the C-20 side chain solvent-exposed. We have previously demonstrated that the monosaccharide ginsenoside-Rh2 is able to potentiate hP2X7 responses (Helliwell et al., 2015) and have proposed a predicted binding model (Bidula et al., 2019b). Ginsenoside-Rh2 and ginsenoside-Rg3 are also predicted to bind in this inverted mode, and both of these ginsenosides can exist as two diasteromers with regard to the C-20 side chain. The natural product form in *P. ginseng* is believed to be the 20(*S*)-diastereomer (Qi et al., 2011; Yang et al., 2014), and this is thought to have higher bioactivity than the 20(*R*)-diastereomer (Wei et al., 2012). We investigated the individual pure diastereomers to determine if this had any bearing on PAM activity at P2X7 and found that only the 20(*S*)-diastereomers of Rg3 and Rh2 are active as PAMs at hP2X7 (Fig. 5). This suggests that the stereospecific positioning of the –OH group on C-20 may be critical for mediating the potentiating effect. To investigate which part of P2X7 would be closest to C-20, we used computational induced-fit docking using Glide as previously reported (Bidula et al., 2019b). The most populated pose for 20(*S*)-Rg3 is presented in Fig. 6, and this places the C-20 hydroxyl group pointing away from the hydrophobic side of the pocket. In an analogous theoretical pose, 20(*R*)-Rg3 would have this C-20 hydroxyl group in close proximity to the hydrophobic face, increasing steric hindrance and repulsive effects plus the desolvation penalty as mentioned above, preventing 20(*R*)-Rg3 and 20(*R*)-Rh2 from interacting with the binding pocket on P2X7.

#### Modulation of Endogenous Human P2X7 in Immune Cells.

**Fig. 7.**
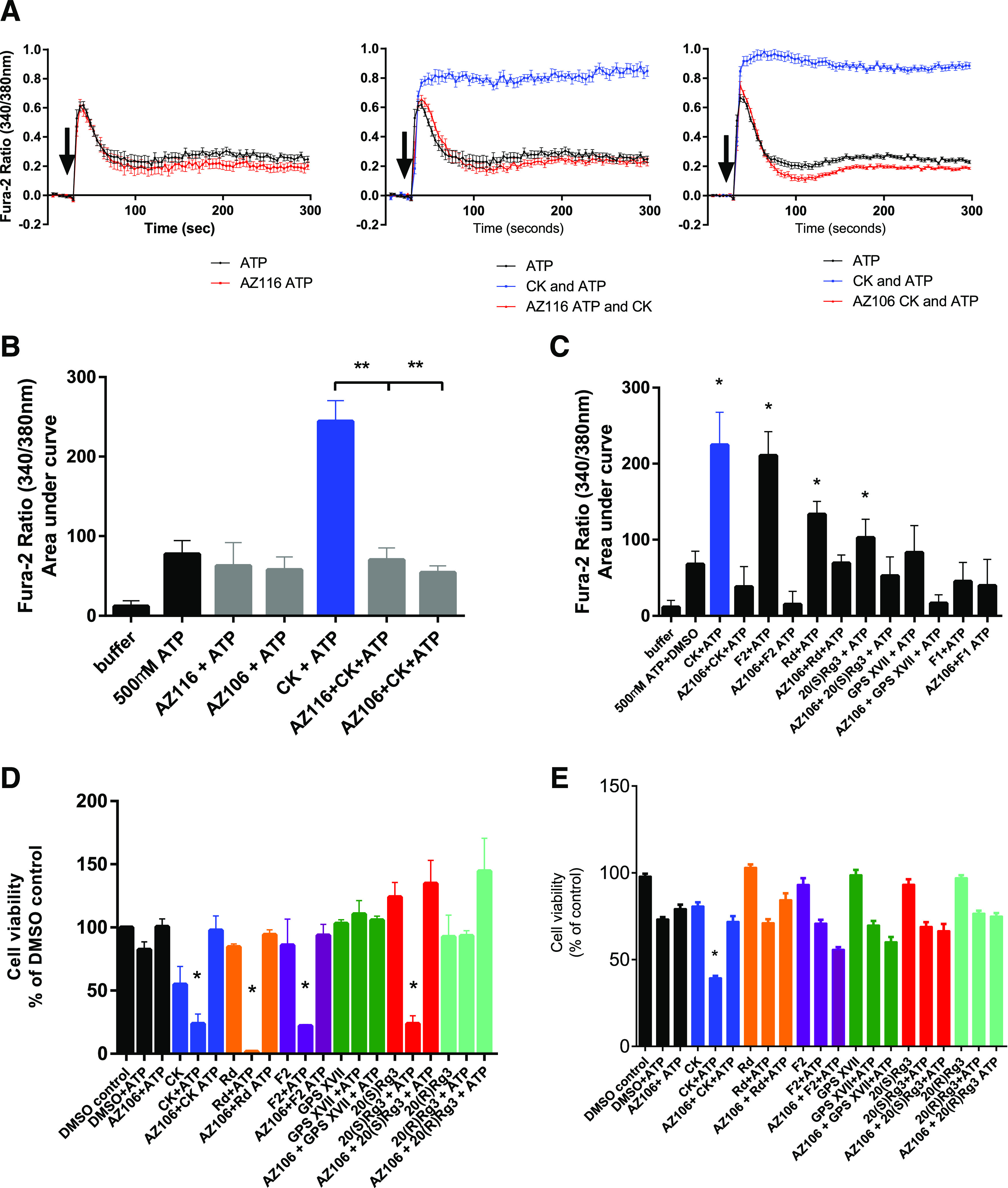
The PAM effects of glycosides in human THP-1 monocyte cell line. (A) ATP-induced calcium responses were measured in fura-2 AM loaded THP-1 cells in suspension using a Flexstation 3 plate reader. Agonist (500 µM ATP) and PAM (ginsenoside-CK 10 µM) were coinjected after establishment of a baseline for 40 seconds. Cells were preincubated with various P2X7 antagonists for 10 minutes prior to start of plate recordings. (B) Summary of collated data from calcium measurements. Fura-2 responses were calculated as area under curve. Data were analyzed using one-way ANOVA with Tukey’s multiple comparisons test to assess the effect of antagonists. *P < 0.05. (C) Investigating the P2X7 dependence of glycoside effects on ATP-induced calcium responses. AZ10606120 (AZ106; 10 µM) was added to cells to block P2X7 receptors prior to measuring calcium responses. Data were analyzed using one-way ANOVA with Dunnett’s multiple comparison test comparing each column against the control (500 µM ATP + DMSO). *P < 0.05. (D) Cell viability experiments were performed over 24 hours using HEK-hP2X7 cell line. Alamar blue fluorescence was measured, and data were normalized to percentage of control (DMSO). Data were collated from five independent experiments. One-way ANOVA was used to analyze the data with Sidak’s multiple comparisons test to compare selected pairs of columns (DMSO + ATP vs. ginsenoside + ATP). *P < 0.05. (E) Cell viability experiments were performed over 24 hours using THP-1 cells. Alamar blue fluorescence was measured and data were normalized to percentage of control (DMSO). Data were collated from five independent experiments. One-way ANOVA was used to analyze the data with Sidak’s multiple comparisons test to compare selected pairs of columns (DMSO + ATP vs. ginsenoside + ATP). *P < 0.05.

Finally, we verified our findings on the identified active versus inactive glycosides by using a human THP-1 monocytic cell line known to endogenously express P2X7 (Fig. 7). Using fura-2 AM loaded cells, we measured ATP-induced calcium responses (Fig. 7A) and found that there was a rapid peak increase in calcium followed by a sustained elevation of calcium over 300 seconds of recording. Pretreatment of THP-1 cells with commercially available P2X7-selective antagonists such as AZ11645373 or AZ10606120 did not dramatically affect the response to 500 µM ATP (Fig. 7), but coapplication of ginsenoside-CK with 500 µM ATP increased the peak response and the sustained elevation in [Ca^2+^]_i_ (Fig. 7). This increased response could be completely abolished in cells pretreated with either AZ11645373 (10 µM) or AZ10606120 (10 µM), suggesting that the ginsenoside-CK potentiated response is solely mediated by P2X7 with no involvement of other purinergic receptors. Using the same protocol we investigated whether we could verify active and inactive glycosides as PAMs and confirmed that ginsenoside-F2, ginsenoside-Rd, and ginsenoside-20(*S*)-Rg3 all potentiated the ATP-induced calcium response in THP-1 cells and that this potentiation was abolished by pretreatment with a P2X7-selective antagonist (Fig. 7C).

We then determined if the identified active PAMs ginsenoside-F2, 20(*S*)-Rg3, and gypenoside XVII could enhance P2X7-dependent cell death in the highly expressing HEK-hP2X7 model using ginsenoside-CK as a positive control. Both ginsenoside-F2 and 20(*S*)-Rg3 could reduce cell viability over 24 hours when applied in combination with ATP, and this was prevented by pretreatment with AZ10606120 (Fig. 7D). However, neither gypenoside XVII nor 20(*R*)-Rg3 in combination with ATP affected cell viability (Fig. 7D). In THP-1 cells, treatment with 500 µM ATP significantly reduced cell viability to 73.3% ± 14.4% of control (Fig. 7E), although this may not be P2X7-dependent due to the lack of effect of AZ10606120. Only ginsenoside-CK could significantly enhance this ATP-induced cell death in THP-1 cells (Fig. 7E).

## Discussion

In summary, we have demonstrated that the chemical requirements for positive allosteric modulators at P2X7 appear to be quite stringent. Dose-response experiments demonstrate that ginsenoside-CK and ginsenoside-Rd have two effects on ATP-induced responses at hP2X7 (Fig. 1B): an increase in the maximum response (a type I PAM effect) and a shift in the dose-response curve to the left (a type II PAM effect), thereby enhancing the maximum effect of the agonist and reducing the EC_50_ value. Our previous work has shown ginsenoside-CK, a triterpenoid glycoside with one glucose attachment, to have the best PAM effect at human P2X7. Here we show that ginsenoside-F2 and ginsenoside-20(*S*)-Rg3 have equivalent PAM activity at P2X7. We investigated a range of glycosides with different numbers of carbohydrate groups attached, and alternative sugar groups to glucose. Of these, gypenoside XVII showed the best PAM activity at P2X7. This glycoside is a dammarene glycoside found in *Panax* species, typically *Panax notoginseng* (Sakah et al., 2013). Gypenoside XVII has a single glucose attached at C-3 and two glucose groups attached at C-20 [a *β*-d-glucopyranosyl-(1–6)-*β*-d-glucopyranoside]. It is very similar in structure to stevenleaf (also known as gypenoside IX; Table 1), but the sugar attachments on C-20 are different. Stevenleaf has a glucose-xylose disaccharide attached at C-20, whereas in gypenoside XVII, this is a glucose-glucose disaccharide. The reduced activity of stevenleaf (Supplemental Fig. 1) compared with gypenoside XVII suggests that there is a preferential requirement for glucose within the binding pocket on P2X7. Similarly, esculentoside A has a similar structure to ginsenoside-20(*S*)-Rg3 with a disaccharide on C-3, although this is composed of glucose-xylose, whereas Rg3 has a glucose-glucose disaccharide. Esculentoside A was inactive at P2X7 (Supplemental Fig. 1).

**Fig. 8.**
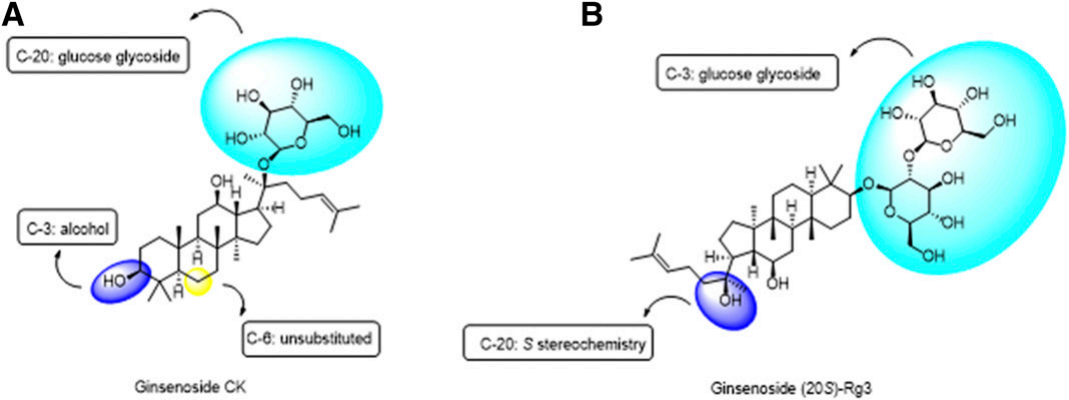
Proposed structure-activity relationship for glycosides acting as PAMs at P2X7. (A) The chemical structure of ginsenoside-CK is shown with important groups highlighted. Glucose attachment (cyan) is critical for activity at P2X7. C-6 substitutions are not tolerated (yellow). (B) The chemical structure of ginsenoside-20(*S*)-Rg3 is demonstrated with positioning relative to the P2X7 binding mode (inverted). The C-3 glucose attachments (cyan) face up into the binding pocket. The C-20 hydroxyl group shows that stereochemistry and positioning are critical for activity at P2X7.

Most useful in terms of defining a structure-activity relationship for PAMs at P2X7 was the finding that modifications at C-6 were not tolerated. Ginsenoside-F1 showed no PAM activity at P2X7, and computational docking suggests that this additional hydroxyl on C-6 faces the hydrophobic side of the predicted binding pocket. This may be incompatible with binding to P2X7 to produce effective potentiation of responses. We have not performed a binding assay to determine if this lack of activity equates to a lack of binding to P2X7 rather than equivalent binding and a lack of effect. Currently there are no suitably labeled ginsenosides available as probes, and this is something we shall explore in future studies. We also discovered that the stereochemistry of 20(*S*) versus 20(*R*) in ginsenoside-Rg3 and ginsenoside-Rh2 have a clear effect on PAM activity with only the 20(*S*) diastereomers retaining good PAM activity. Again, computational docking suggests this C-20 hydroxyl group to be close to the highly hydrophobic side of the predicted binding pocket, and we hypothesize that this is important in correct positioning leading to effective potentiation of ATP-dependent responses. Regarding the differences in the terpenoid backbone, several of the compounds we selected for testing contained carbohydrate at either end of the molecule but did not contain the dammarene scaffold. For example, daucosterol is a monosaccharide (glucose) but contains a sitosterol-like scaffold. This includes a double bond that changes the shape of the scaffold and this has a detrimental effect on activity at P2X7. The key elements to the structure-activity relationship for glycosides at P2X7 are summarized in Fig. 8 showing two predicted binding modes for glycosides. Figure 8A shows the ginsenoside-CK binding mode with C-20 glucose inserted into the binding pocket. Figure 8B shows the inverted mode for ginsenosides with C-3 glucose inserted into the binding pocket. Substitutions are tolerated on C-3 and C-20, but glucose is the preferred sugar attachment. Monosaccharides have higher activity than most disaccharides, and no substitution is tolerated at C-6 (Fig. 8A). In the inverted mode, the stereochemistry of the hydroxyl group on C-20 is critical for activity (Fig. 8B). In both cases the dammarene scaffold is favored over a sitosterol scaffold.

It is important to perform full dose-response analysis to determine those compounds that have PAM effects at P2X7. Saikosaponin A and solanine could increase the ATP-induced dye uptake in the screen on HEK-hP2X7 cells (Fig. 2A), but further investigation showed that these compounds had a toxic effect on the cells, inducing YO-PRO-1 dye uptake in the absence of ATP or the absence of the P2X7 receptor (Fig. 2). Saikosaponins have been linked to multiple biologic effects including induction of apoptosis (Li et al., 2018).

Several groups have demonstrated endogenous steroidal compounds to have positive allosteric modulator activity at P2X receptors. Dehydroepiandrosterone and progesterone can potentiate rodent P2X2 receptors (De Roo et al., 2003, 2010), and 17*β*-ester derivatives of testosterone also potentiate rat P2X2 and P2X4 (Sivcev et al., 2019). None of these compounds had activity at P2X7 receptors. Lithocholic acid, a bile acid, has potentiating activity at P2X7 and P2X4 (rat) but inhibits rat P2X2 (Sivcev et al., 2020). This suggests there may exist endogenous regulators of P2X channel activity. It has been suggested that the bile acids may share a similar binding site to ivermectin, close to transmembrane domain 1 (Sivcev et al., 2020).

Positive allosteric modulation of P2X7 may be important in a number of contexts (Stokes et al., 2020). In this study we used the human monocytic cell line, THP-1, which has been used in other studies to investigate human P2X7 responses (Stokes et al., 2006; Gadeock et al., 2010). Native P2X7 responses are small in this cell line, likely masked by the multitude of other purinergic ATP-responsive receptors present. P2X7-selective antagonists did not affect the ATP-induced calcium responses dramatically (Fig. 7A), yet we did see robust potentiation of the ATP-induced calcium response that was mediated by P2X7. The THP-1 cell line carries a loss-of-function polymorphism in the C-terminus of P2X7, rs3751143, encoding the Glu496 > Ala amino acid substitution. Sequencing of exon 13 reveals heterozygosity for rs3751143 (Supplemental Fig. 2), confirming the work in Gadeock et al. (2012). Our data clearly show that P2X7 responses in THP-1 cells can be enhanced by ginsenoside-CK, and we can effectively rescue deficient P2X7 receptor responses in humans carrying this loss-of-function polymorphism. Ginsenoside-F2 and ginsenoside-Rd were also effective at increasing P2X7 responses in THP-1 cells (Fig. 7), and ginsenoside-F1 was inactive. Extending this set of data that measures immediate P2X7 responses, we looked at P2X7-mediated cell death first in HEK-hP2X7 cells using an Alamar blue cell viability assay. Ginsenoside-CK, ginsenoside-Rd, 20(*S*)-Rg3, and ginsenoside-F2 were very effective at enhancing cell death in combination with ATP; however, gypenoside XVII was unable to enhance P2X7-mediated cell death in this cell line (Fig. 7D). At this stage, it is unclear why this would be the case. Future investigations into the longevity of the potentiation may reveal that gypenoside XVII has a shorter duration of action than the ginsenosides, perhaps due to differential binding modes. Repeating this work in THP-1 cells showed that only ginsenoside-CK was effective at potentiation of cell death induced by ATP (Fig. 7E). This may be due to the lower level of expression of P2X7 in THP-1 cells, the expression of other purinergic receptors that may bind ginsenosides such as P2X4, or simply due to duration of action. It is also conceivable that monocytes may release factors such as glycosidase enzymes that can degrade ginsenosides.

Other compounds can act as positive allosteric modulators of P2X7 (Stokes et al., 2020), including clemastine (Nörenberg et al., 2011), tenidap (Sanz et al., 1998), ivermectin (Nörenberg et al., 2012), and polymyxin B (Ferrari et al., 2004). These bear no structural similarities to ginsenosides, although ivermectin is a glycoside, containing two oleandrose sugars. As yet nothing is known about the potential site of action of ivermectin on P2X7, although on P2X4 the binding site is proposed to be close to transmembrane domain 1 (Asatryan et al., 2010, Samways et al., 2012). In terms of therapeutic relevance of PAMs acting at P2X7, most exciting is the potential to enhance the microbicidal activity of immune cells. P2X7 has been implicated in regulation of pathogen killing, particularly those residing in intracellular locations such as mycobacteria and parasites (Di Virgilio et al., 2017, Savio et al., 2018). The mechanisms by which P2X7 contributes to reduction of pathogen burden are not yet well understood but could involve cytokine/mediator secretion, reactive oxygen species generation, or host cell apoptosis. We recently showed that potentiation of P2X7 responses with ginsenoside-CK changed the type of cell death in the J774 macrophage cell line compared with high concentrations of ATP (Bidula et al., 2019a), and this could be relevant within an immune response. In models of infection, most of the work has been performed on P2X7-deficient mice to understand the role of this receptor within the host immune response. However, there has not been an in vivo study to investigate pharmacological targeting of P2X7 with a PAM. This idea has been tested in a zebrafish whole-animal study for *Mycobacterium marinum* infection using clemastine to potentiate P2X7 (Matty et al., 2019), providing evidence that targeting P2X7 may be beneficial to control mycobacterial infections.

## Abbreviations

AM: acetoxymetyl ester
CK: compound K
DMEM: Dulbecco’s modified Eagle’s medium
hP2X7: human P2X7
HEK-hP2X7 cell: HEK-293 cell stably expressing the wild-type hP2X7 variant
PAM: positive allosteric modulator
PPD: protopanaxadiol
